# Endoscopic ultrasound-guided antegrade treatment with uncovered self-expanding metal stent for malignant afferent loop syndrome-complicated cholangitis after biliary reconstruction

**DOI:** 10.1055/a-2313-3923

**Published:** 2024-06-05

**Authors:** Yoshinori Shimamoto, Hirotsugu Maruyama, Tatsuya Kurokawa, Yuki Ishikawa-Kakiya, Kojiro Tanoue, Akira Higashimori, Yasuhiro Fujiwara

**Affiliations:** 1 Department of Gastroenterology, Osaka Metropolitan University Graduate School of Medicine, Osaka, Japan

Malignant afferent loop syndrome often causes cholangitis and jaundice 1 2 , necessitating treatment. Endoscopic treatment is minimally invasive and utilizes a natural orifice, proving advantageous surgical or percutaneous management. Endoscopic ultrasound (EUS)-guided gastrojejunostomy (EUS-GJ) 3 4 is efficacious; however, severe adverse events are a concern. Therefore, treatment using physiological orifices is desirable. Herein, we report the first case of successful uncovered self-expanding metal stent (USEMS) placement with EUS-guided antegrade treatment using a physiological orifice for malignant afferent loop syndrome after biliary reconstruction for cholangiocarcinoma.

A 76-year-old woman who had undergone chemotherapy for peritoneal dissemination recurrence after biliary reconstruction and total pancreatectomy for distal cholangiocarcinoma and main pancreatic duct-type intraductal papillary mucinous neoplasm was admitted to our hospital for cholangitis. Contrast-enhanced computed tomography revealed afferent loop dilation; however, we suspected choledochojejunostomy-associated stenosis due to peritoneal dissemination ( Fig. 1 ) and planned EUS-guided hepaticogastrostomy (EUS-HGS) ( Video 1 ).

**Fig. 1:**
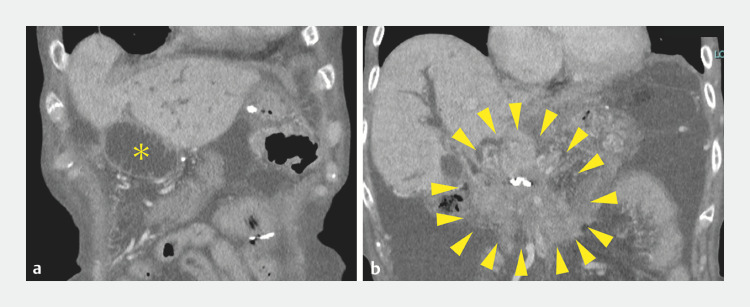
**a** Contrast-enhanced computed tomography on admission showed dilatation of afferent loop (yellow asterisk). **b** Peritoneal dissemination near choledochojejunostomy.

Successful endoscopic ultrasound-guided antegrade treatment using uncovered self-expanding metal stent for malignant afferent loop syndrome-complicated cholangitis due to tumor recurrence after biliary reconstruction.Video 1

First, B3 puncture was performed using a 22-gauge needle and a 0.018-inch guidewire followed by double-lumen catheter insertion (Uneven Double Lumen Cannula; Piolax Medical, Kanagawa, Japan). Contrast injection revealed bilateral hepatic ductal dilation; however, no stenosis was observed at the choledochojejunostomy. Thereafter, the guidewire and catheter were advanced into the jejunum; contrast injection revealed stenosis of the afferent loop. We diagnosed cholangitis complicated by malignant afferent loop syndrome due to peritoneal dissemination and cholangiocarcinoma recurrence. A guidewire was advanced across the stenosis, AND a 10-mm USEMS (YABUSAME Neo; Kaneka Co., Tokyo, Japan) was successfully placed in the afferent loop stenosis ( Fig. 2 ). After the USEMS placement, the contrast injection passed satisfactorily, and no adverse events were observed.

**Fig. 2:**
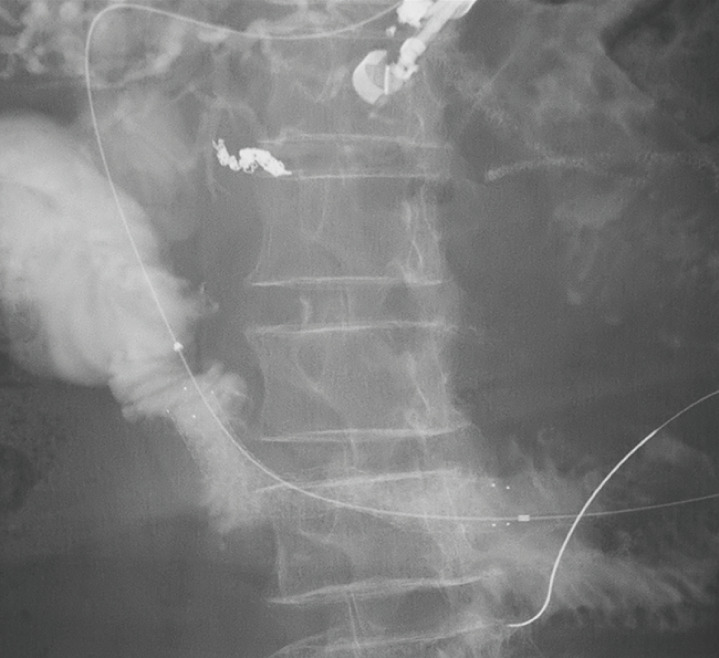
Successful uncovered self-expanding metal stent placement with ultrasound-guided antegrade endoscopic treatment for malignant afferent loop syndrome after biliary reconstruction for cholangiocarcinoma.

This method involves treatment through a physiological orifice, which raises fewer concerns about adverse events than those associated with EUS-GJ and is more physiological than EUS-HGS. Furthermore, it permits approaching the intestinal tract, which cannot be visualized using EUS. This technique may be a novel treatment strategy for malignant afferent loop syndrome.

Endoscopy_UCTN_Code_TTT_1AS_2AG

## Notes

Endoscopy E-Videos https://eref.thieme.de/e-videos
*E-Videos* is an open access online section of the journal *Endoscopy* , reporting on interesting cases and new techniques in gastroenterological endoscopy. All papers include a high-quality video and are published with a Creative Commons CC-BY license. Endoscopy E-Videos qualify for HINARI discounts and waivers and eligibility is automatically checked during the submission process. We grant 100% waivers to articles whose corresponding authors are based in Group A countries and 50% waivers to those who are based in Group B countries as classified by Research4Life (see: https://www.research4life.org/access/eligibility/ ). This section has its own submission website at https://mc.manuscriptcentral.com/e-videos .
